# Trends in Managing Cardiac and Orthopaedic Device-Associated Infections by Using Therapeutic Biomaterials

**DOI:** 10.3390/polym13101556

**Published:** 2021-05-12

**Authors:** Stefania Scialla, Giorgia Martuscelli, Francesco Nappi, Sanjeet Singh Avtaar Singh, Adelaide Iervolino, Domenico Larobina, Luigi Ambrosio, Maria Grazia Raucci

**Affiliations:** 1 Institute of Polymers, Composites and Biomaterials of National Research Council (IPCB-CNR), 80125 Naples, Italy; stefania.scialla@cnr.it (S.S.); domenico.larobina@cnr.it (D.L.); 2 Multidisciplinary Department of Medical-Surgical and Dental Specialties, University of Campania Luigi Vanvitelli, 81100 Naples, Italy; martuscelligiorgia@gmail.com; 3 Centre Cardiologie du Nord de Saint-Denis, Department of Cardiac Surgery, 93200 Paris, France; f.nappi@ccn.fr (F.N.); adelaide.iervolino01@icatt.it (A.I.); 4 Department of Cardiothoracic Surgery, Golden Jubilee National Hospital, Glasgow G81 4DY, UK; sanjeetsa_singh@gmail.com

**Keywords:** cardiac-associated infections, orthopaedic-associated infections, biofilm, passive antifouling strategies, active antimicrobial strategies, antimicrobial peptides, ionic liquids

## Abstract

Over the years, there has been an increasing number of cardiac and orthopaedic implanted medical devices, which has caused an increased incidence of device-associated infections. The surfaces of these indwelling devices are preferred sites for the development of biofilms that are potentially lethal for patients. Device-related infections form a large proportion of hospital-acquired infections and have a bearing on both morbidity and mortality. Treatment of these infections is limited to the use of systemic antibiotics with invasive revision surgeries, which had implications on healthcare burdens. The purpose of this review is to describe the main causes that lead to the onset of infection, highlighting both the biological and clinical pathophysiology. Both passive and active surface treatments have been used in the field of biomaterials to reduce the impact of these infections. This includes the use of antimicrobial peptides and ionic liquids in the preventive treatment of antibiotic-resistant biofilms. Thus far, multiple in vivo studies have shown efficacious effects against the antibiotic-resistant biofilm. However, this has yet to materialize in clinical medicine.

## 1. Introduction

The use of orthotics (cardiac pacemakers, defibrillators, and stents) and prosthetics (heart valves, fracture fixation, joint prostheses) medical devices has grown exponentially over the past half-century, ameliorating patients’ quality of life (QoL). More than 1.7 million cardiovascular devices and over 1 million orthopaedic prostheses are implanted worldwide annually [1,2], yielding a global implants market of $21.5 USD [3] and $55.8 billion USD [2] in 2020, respectively. It is expected to increase further in the near future.

Metallic biomaterials have been enormously used in orthotic and prosthetic medical devices. Nowadays, stainless steels, titanium (Ti6Al4V), and cobalt alloys are often used as plates, screws, and pins for fixation, tooth implants, or coronary stents [4,5], owing to their high strength and stiffness properties, along with their corrosion resistivity and inherent biocompatibility [6]. However, also ceramics (i.e., silicates glasses, hydroxyapatite (HA)) and polymeric materials (i.e., silicone elastomers, polycaprolactone (PCL), polyglycolic acid (PGA), polylactic acid (PLA), polyetheretherketone (PEEK)) and their composite [4] have been used in orthopaedic implants.

Although indwelling medical devices are to date implanted in nearly all anatomical districts of the body, they are anyway “foreign bodies”. Therefore, these foreign materials may trigger a local immune-compromised environment (*locus minoris resistentiae)* in the host and provide fertile conditions for biofilm production and for the resultant implantable medical device-associated infection (MDI) onset [7].

MDIs represent nearly 50–70% of the 2 million healthcare-associated infections (i.e., infective endocarditis, osteomyelitis, prosthetic joint infections). MDI management involves lengthy and costly treatments (i.e., long-term hospitalization, multiple risky surgeries, and secondary complications), remarkably burdening throughout the healthcare system [8]. Despite advances in biomaterials and surgical techniques, cardiac and orthopaedic device-associated infections (cDAIs and oDAIs, respectively) remain an unmet clinical complication. In fact, the risk of infection depends on the type of device, its level of invasiveness in the body, the anatomical site of insertion, and the duration of the implant (transient or permanent). In addition, selective diagnostic criteria able to distinguish MDI from the failure of an implant remaining sterile, as well as the timely choice of proper antibiotics or surgical methods for treatment, remain controversial.

Preventive measures aimed at cDAIs and oDAIs managing should be focused primarily on discouraging biofilm formation while ensuring the functional activity of host cells for suitable implant integration. Conservative treatments of MDIs aim at biofilms eradication through a severe systemic antibiotic therapy (6–12 weeks) to save the implant [9]. However, the presence of antibiotic-resistant bacteria, weak drug bioavailability, and absorption at the infection site are the main limiting issues of conservative MDI therapies. If the infection takes hold, invasive and highly risky revision surgeries are inevitable [10,11].

To overcome these limitations, several effective antifouling and antimicrobial strategies [12] have been proposed over time and classified in:

- Passive antifouling surface modifications;
- Active antimicrobial surface modifications;
- Peri-operative antimicrobial local carrier and coatings [13].

Passive and active antimicrobial strategies aim at thwarting adhesion and maturation phases of biofilm formation by:

- Modifying implants’ surface chemistry (i.e., wettability, surface energy, potential, and conductivity);
- Modifying implants’ surface topography (i.e., crystallinity, roughness);
- Functionalizing implants’ surface with bactericidal agents-loaded coatings (i.e., metal ions, antiseptics or organic molecules, antibiotics) [13].

However, the lack of a universal surface treatment suitable for any microbial strain and implant; a short-lasting (i.e., over 2 weeks) and microbial strain-dependent antifouling feature; along with difficult-to-predict long-term effects after antibacterial coatings depletion, are some of the issues limiting the efficacy antimicrobial passive and active implants’ surface modifications in clinical practice.

Comparing to the existing reviews, the present work stands out for reporting the last 5-years advancements in implants’ surfaces antimicrobial modification strategies, by highlighting the dual antimicrobial and regenerative potential of promising therapeutic biomaterials (i.e., ionic liquids), in the framework of managing antibiotic-resistance biofilm in cardiac and orthopaedic indwelling devices-associated infections.

## 2. Causative Agents Involved in Cardiac and Orthopaedic Device-Associated Infections

The human body is inhabited by a multitude of commensal bacteria, establishing a positive symbiotic relationship with the host (i.e., saliva, gastrointestinal tract, oral cavity, ear canal, mucosa, and skin), helping in several metabolic activities and innate defence mechanisms against pathogens [14]. Deregulation of host-commensal bacteria homeostasis in the presence of a foreign body, such as an indwelling implant, may result in a pathogenic biofilm formation and causing the onset of MDIs. Among the several microbial strains involved in the MDIs, there are [15]: Gram-positive (Enterococci, Staphylococci, and Streptococci), Gram-negative (*Klebsiella*, *Pseudomonas*, and *Enterobacter*), as well as fungi (*Candida albicans*) and yeasts (*Cryptococcus*, *Trichosporon*, and *Saccharomyces*) [16]. In Table 1 are listed the main microbial strains involved in the cDAI and oDAI. *S. aureus* represents the most common pathogen among healthcare-associated infections, accounting for around 30% of cases [17].

### 2.1. Cardiac Device-Associated Infections

Infective endocarditis (IE) caused by *S. aureus* bacteraemia accounts for 70% of total cases. *S. aureus* endocarditis is extremely aggressive and leads to an increased risk of embolism, stroke, persistent bacteraemia, and death [17,19]. In high-income countries, oral Streptococci can account for about 20% of cases, while Enterococci are responsible for a further 10% [19]. A very serious threat comes from infections caused by coagulase-negative Staphylococci (CoNS) (i.e., *S. epidermidis*, *S. lugdunensis*, and *S. capitis*), which are ubiquitous pathogens and skin commensals. They colonize infusion catheters and permanent devices but are also the most common cause of early IE developed in biomaterials constituting valve bio-prostheses [24]. The percentage of infection caused by CoNS can reach up to 10% of the infectious colonization in implanted biomaterials, thus playing an important role in cDAI [25]. These early-onset infections occur immediately after the first surgical implantation, within the first year. Furthermore, the colonization of infection foci by methicillin-resistant *Staphylococcus lugdunensis* strain is a cause of great concern due to its particular aggressiveness toward biological tissues and biomaterials [25]. A combination of opportunistic, zoonotic bacteria and fungi may cause particularly insidious infections. Bacteria belonging to the HACEK group (*Haemophilus*, *Aggregatibacter*, *Cardiobacterium*, *Eikenella corrodens*, *Kingella*), although infrequent (accounting for only 3% of cases), are slow-growing organisms that colonize the oropharynx and can adhere to cardiac devices of immunosuppressed patients. Rare pathogens include Gram-negative bacteria (i.e., *Acinetobacter* spp., *P. aeruginosa*, *Legionella* spp., *Mycoplasma* spp. and *Tropheryma whippelii*) [17,19]. Infections caused by fungi (i.e., *Candida* or Aspergillus), although less common, are often fatal. They arise especially in immunosuppressed patients or post-cardiac surgery, especially in those undergoing implantations of prosthetic valves or devices for the treatment of arrhythmias [17,19].

#### 2.1.1. Infections in the New Cardiac Platform

Transcatheter heart valve (THV) prostheses, i.e., balloon, self, and mechanical expandable systems, are subjected to specific infections. A list of articles evaluating the development of infection in patients undergoing balloon or self-expandable TAVR is reported in Table 2. A very low rate of infection was described in the pilot PARTNER randomized trial [26,27]. The landmark evaluation for the rate of infection after the TAVI procedure was a multicenter study from 47 centers worldwide that revealed 250 cases of IE in recipients of self and balloon-expandable devices. The overall incidence was 1.1% per person-year at a median of 5.3 months post-procedure. The causative pathogens that spread on the surface of biomaterial and frame stent were Enterococci strains in 24.6% and *S. aureus* in 23.3%. The in-hospital mortality was higher, with a rate of 36%, and 2-year mortality was 67% [28].

Colonization of the device by pathogens was indifferently localized on the stent frame, the leaflets, or both components. It should be noted that antibiotic prophylaxis was used in 59% of the infected and that although the self-expanding CoreValve System (Medtronic, Minneapolis, MN) was an independent risk factor for IE (hazard ratio [HR]: 3.1; CI 95%: 1.37 to 7.14), this deserves further evaluation for validation. None of the materials assembled in the devices described [27,29,30,31,32,33,34] were exempted from the possibility of manifesting an infectious process.

#### 2.1.2. Cardiac Implantable Electronic Devices

Cardiac device-associated infections (cDAI) may occur after the implantation of cardiac implantable electronic devices (CIEDs) that encompasses permanent pacemakers, implantable cardioverter defibrillators, and cardiac resynchronization therapy devices. cDAIs have two main effects. First, infected materials, if not quickly removed, can favour the development of devastating infections with a considerable increase in mortality and morbidity in the short and long term. Second, infections lead to an incremental cost of ownership that has been calculated at more than $15,000 USD per patient [35,36,37].

CDI can be generated by the device and extend to the generator pocket or involve the generator leads. The most frequent and dangerous evolution is the extension of the infectious process to the valvular and non-valvular endocardial surfaces. It is not uncommon that initial inflammatory processes develop, such as cellulitis or erythema, to evolve as a widespread infection involving the materials of the device whose eradication is very difficult. A solution of continuity often occurs between the infected material and an evident erosion of the skin overlying the pocket. Infection of CIED material due to lead colonization through the bloodstream is not uncommon. In this case, the Staphylococci belonging to the CoNS strain represent 60% to 80% of the causative pathogens. Streptococci are the most frequent microorganisms in patients who have cancer of the digestive system and who need the implantation of a CIED [25]. In this case, the gateway for bacteria to enter the blood via the vena cava [37,38]. Today the only solution to CIEDs is the use of antibiotic therapy as prophylaxis, which is evident in both RCTs and observational studies. Prolonged use of antibiotic administration and serial negative blood cultures for 72 h is required before re-implantation if the use of a new device is deemed necessary [39].

**Table 2:** Studies evaluating infection in balloon and self-expanded TAVR.

First Author/ Year/ Type of Study	Total Number	TAVR Model	Type of Material	No. of TAVR-IE Patients	Yr. Incidence of TAVR-IE	Microbiology	Finding	Ref
Makkar 2020 Lancet RCT	750	^†^ Portico, * SAPIEN, SAPIEN XT, SAPIEN 3, * CoreValve, Evolut-R, Evolut-PRO	Porcine-L/Nitonol-S Bovine-L/CrCo-S	-	-	-	Not designed to detect endocarditis	[29]
Lanz 2019 Lancet RCT	739	γACURATE neo, SAPIEN 3	Porcine-L/Nitinol-S Bovine-L/CrCo-S	3 at 30 days	NA	NA	Similar rates of IE between ACURATE neo and SAPIEN 3. Superiority of SAPIEN 3 for early safety and efficacy	[32]
Mack 2019 NEJM RCT	496	SAPIEN 3	Bovine-L/CrCo-S	-	-	-	Not designed to detect endocarditis	[30]
Regueiro 2016 JAMA Retrospective	20006	CoreValve System SAPIEN	Porcine-L/Nitinol-S Bovine-L/CrCo-S	250 at 1 y	1.1%	*Enterococci* (24.6%), *S. aureus* (3.8%), *CoNS*	IE associated with younger age, male sex, history of diabetes, moderate to severe residual aortic regurgitation IE patients had high rates of in-hospital mortality and 2-year mortality	[28]
Mangner 2016 JACC OS	1820	CoreValve System	Porcine-L/Nitinol-S	55 at 1 y	1.82%	*CoPS* (38.2%), *MRSA* and *Enterococci* (30.9%), *CoNS* (9.1%)	Patients in chronic hemodialysis at highest risk group for development and death by IE. Poor prognosis of IE patients	[40]

Abbreviations: ACCURATE neo = self-expandable heart valve; CrCo = chromium-cobaltum; RCT = randomized clinical trial; CoreValve System = self-expandable transcatheter heart valve; OS = observational study; PORTICO IDE = the Portico Re-sheathable Transcatheter Aortic Valve System U.S. Investigational Device Exemption trial; TAVR = transcatheter aortic valve replacement; SAVR = surgical aortic valve replacement; * CoreValve System included CoreValve, Evolut-R, Evolut-PRO (Medtronic, Minneapolis, MN, USA). * SAPIEN included SAPIEN, SAPIEN XT, and SAPIEN 3 (Edwards Lifesciences Irvine, CA, USA); χnot specified which of SAPIEN family. ^†^ PORTICO (Abbott Structural Heart, St Paul, MN, USA). γACURATE neo (BostonScientific, Marlborough, MA, USA).

## 3. Orthopaedic Device-Associated Infections

Orthopaedic device-associated infections (oDAIs) are one of the major early postoperative complications in prosthetic surgery, usually occurring within 3 months after surgery. Osteomyelitis (OM) and prosthetic joint infections (PJI) are severe and deep bone infections that may arise from different routes: bacteraemia, spreading from nearby tissue, or following injury, surgery, or implantation of a foreign body. They share a common spectrum of etiological agent strains, mainly Gram-positive, such as Staphylococci (10–40%) with CoNS (20–40%), and Enterococci (3–7%); even if an increase in infections caused by Gram-negative, such as *Pseudomonas* (≈6%) and *E. coli* (<3%), has been described in recent years [20,21]. OM is a microbial-triggered bone inflammation that simultaneously causes bone and medullar cavity destruction [41], affecting about 2 per 10,000 people [42].

Joints replacement (e.g., hip, knee, shoulder, or elbow arthroplasty) is a well-established clinical procedure worldwide, which restores the anatomical function with life-enhancing benefits for the patient. However, joints replacement may fail due to bone-cement interface loosening, peri-prosthetic fracture, fracture of the prosthetic material itself, wear, implant misplacement, dislocation-instability, or materials fatigue [43]. PJI has 1–9% infection rates, which varies with years after surgery and implantation site. PJI occurs less frequently (0.5–5%) than OM and IA. In the first 2-years, the infection rate is 0.3–1.7%, 0.5–2%, and 2–9% after hip, knee, and elbow arthroplasty, respectively [43]. After surgical revision, infection rates tend to considerably increase (up to 40%) than after primary replacement

Peri-implantitis (PI) is an inflammatory process affecting the surrounding tissues supporting the osseo-integrated dental implants, with a consequent loss of the “implant tooth”. Gram-negative anaerobe bacteria, such as *Porphyromonas gingivalis*, *Aggregatibacter actinomycetemcomitans*, *Bacterioides forsythus*, *Treponema denticola*, *Prevotella nigrescens*, *Peptostreptococcus micros*, and *Fusobacterium nucleatum* are among the main microorganisms associated with peri-implantitis [44]. The creation of the biofilm on the surface of the dental implant begins around 30 min after grafting. Its adhesion and subsequent creation of the biofilm is favoured and facilitated by a layer of proteins and sugars deriving from saliva called “acquired film” (AP), which acts as a buffer between the surface of the implant and bacteria of the oral microbiome. During biofilm formation, a decrease in the levels of *Streptococcus intermedius* is followed by an increase in the pathogen *Eubacterium nodatum*, as a precursor to triggering the infection accompanied by variable host factors such as uncontrolled diabetes mellitus, autoimmune disorders, genetic component, smoking, bacterial contamination and alterations in the dental status. Studies have reported infection-related implant loss in 20% of patients during 5–10 years after implant placement [44].

Cochlear implants are also prone to infections caused by upper respiratory tract pathogens, such as *S. aureus*, *S. pneumoniae*, *Haemophilus influenzae*, or *Moraxella catarrhalis* [23]. These pathogens are typically implied in cochlear implant infections, including postoperative wounds (1–12%), otitis media (OTM), and bacterial meningitis [23]. Particularly in children with cochlear implants, acute otitis media may cause the inner-ear infection, leading to hearing loss along with implant failure and even meningitis [23].

## 4. Biofilm Formation Process

Bacteria may exist in a planktonic state (free-floating) and sessile state (adhered to a surface), exhibiting very distinct features [45]. Planktonic bacteria rapidly multiply and have high motility. Therefore, they are more susceptible to the effects of antibiotics, environmental (i.e., UV light, desiccation, heat, cold, shear forces), and host factors. Conversely, sessile bacteria grow very slowly on surfaces for nutrient limitations and limited mobility. However, they can elude antagonistic factors by forming aggregates, altering their physiology, and taking advantage of deficiencies in the host clearance mechanisms to cause infection [45].

Biofilms are sessile, self-structured and autonomously replicating microbial communities embedded in an inhomogeneous and self-produced extracellular matrix (ECM), which provides water, nutrients, and oxygen availability for cell sustenance and growth, as well stability and protection of the biofilm [45]. Microbial communities’ lifestyle inside of biofilms is the result of a deep transformation of the microbes’ physiology and metabolism, which has led to peculiar tolerance against environmental or xenobiotic stresses and the host’s immune system. Biofilm provides an ever-growing niche for microorganisms, allowing them to access the blood circulation and deep tissues, as well spread to other body sites. Biofilm formation, also called bio-fouling, is the result of “the race for the surface” [20] between host tissue cells and microbial colonization of the implant’s surface, which dictate integration or rejection fate.

**Figure 1:**
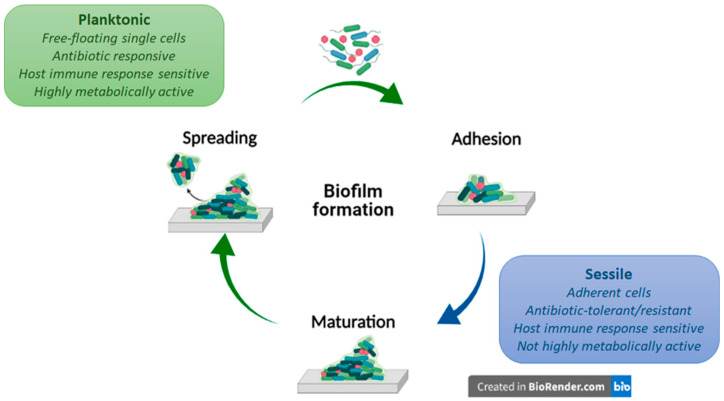
Schematic representation of a biofilm formation process. Biofilm formation is a life-cyclic process in which microbial cells take turns with their planktonic and sessile lifestyle. The whole process involves an early reversible interaction between planktonic cells, which tend to strengthen and form a monolayer irreversible attached to the surface (1). Irreversibly attached bacteria start producing an EPS matrix, splitting in multicellular growing microcolonies and turning into a mature biofilm (2). Growth-limiting conditions trigger biofilm spreading (3), causing infections and/or colonizing a new surface. Created with BioRender (https://biorender.com/ (accessed on 1 March 2021)).

Biofilm formation on the implant’s surface is a finely tuned and life-cyclic process, in which microbial cells start and revert to their planktonic lifestyle, evolving in the middle into a sessile surface-attached state favourable for microbial colonization. Few single cells initially get in touch with a material’s surface, engaging weak and reversible interactions, which tend to strengthen and yield an irreversible adhesion (1). Irreversibly attached cells start the material’s surface colonization, splitting in multicellular growing microcolonies (2) and turning into a mature biofilm. As the biofilm matures, microcolonies may undergo growth-limiting conditions, which trigger their spreading from the biofilm (3), causing infections and/or colonizing a new surface. A scheme of the biofilm formation process is reported in Figure 1.

### 4.1. Bacteria Adhesion

Initial planktonic bacteria adhesion to biomaterials surface results from attractive and repulsive forces, which are reversible and non-specific (i.e., London—van der Waals, electrostatic attraction forces, and acid-base hydrophobic interactions), acting at long-range distances (>50 nm) [46]. By contrast, permanent adhesion is triggered by irreversible and specific interactions, which act at short-range interactions (<5 nm) [20,46], and by the activation of small signalling molecules expression (i.e., cyclic-di GMP or non-coding small RNAs). Cyclic-di GMPs regulate the extracellular polysaccharide adhesins expression, resulting in extracellular appendages production (i.e., pili, fimbriae, and pilus-like adhesion structures) [46]. Through them, the bacteria body is reoriented from a polar to a longitudinal attachment, switching from a free-living to a sessile lifestyle [46]. Non-coding small RNAs regulate adhesion via the post- transcriptional control of adhesion genes, such as those required for exopolysaccharide production [46].

Furthermore, adhesive components of hosts’ ECM (i.e., fibrin, platelet microthrombi, fibronectin, fibrinogen, vitronectin, laminin, collagen (Coll), von Willebrand factor, and polysaccharides) promote microbial attachment and further colonization of a biomaterial surface through a thrombosis onset [47]. During exposure of an implant surface to human blood plasma, there is rapid surface adsorption of plasma proteins [47].

### 4.2. Biofilm Maturation

Once irreversible bacteria adhesion is achieved, the colonization of the surface and the establishment of a mature multicellular biofilm may start. Initially, attached bacteria organize in a monolayer enveloped by a protective extracellular matrix, 0.2–1.0 μm thick [17]. Generally, the bacteria represent 5–35% of biofilm volume; while the remaining volume consists of extracellular polymeric substances (EPS) involving exopolysaccharides (1–2%), structural proteins (>2%), cell debris, and extracellular nucleic acids (eDNA < 1%), *ions*, teichoic and lipoteichoic acids, and water (97%) [17]. Polysaccharide biofilm production sustains both bacterial persistence and resistance to antibiotics [47]. P. aeruginosa produces alginate, cationic (Pel), and neutral (Psl) exopolysaccharides [48], while S. epidermidis and *S. aureus* produce polysaccharide intercellular adhesin (PIA) [49], conferring higher resistance to antibiotics, such as aminoglycosides [20]. eDNA is crucial in stabilizing and strengthening biofilm matrix; supplying the nutrients, modulating the mechanisms underlie biofilm susceptibility or resistance to antibiotics [20]. EPS accumulation promotes bacterial microcolonies formation and also sustains their growth in three-dimensional structured communities (10–30 nm thick) [17], owing to a sophisticated quorum-sensing system, which modulates their behaviour in response to external stimuli (i.e., stress and cell density) [50]. Quorum-sensing system is a signalling pathway, where bacterial microcolonies use autoinducers molecules (i.e., acyl-homoserine lactones in Gram-negative and oligopeptides in Gram-positive) to communicate among them, detect the presence of other cells, and appropriately trigger the expression of specific genes [51]. These may affect the structure of the colony, select for the growth of a specific strain, and lead to antibiotic resistance [51]. For example, *S. aureus* quorum-sensing system is encoded by *Agr*, which regulates the production of virulence factors in biofilm-associated infections, such as endocarditis and osteomyelitis [20].

### 4.3. Biofilm Spreading

As the biofilm matures, nutrients and oxygen become limited, accumulating toxic products. These stress-inducing conditions push mature microbial macrocolonies to detach from the biofilm, revert to a planktonic state, and spread in other regions of the host’s body or the implant, causing infections. This process is often referred to as metastatic seeding [52]. The detachment phase is characterized by concomitant expression of different saccharolytic enzymes (i.e., N-acetyl-heparosan lyase, alginate lyase, hyaluronidase, β-lattamasi, etc.), which help the attached microbial colonies to release from the surface; and along with adhesins, extracellular appendages up-regulation let the bacteria move toward a new site [17].

### 4.4. Antibiotic Resistance in Biofilms

Bacteria biofilms involve highly dense and genetically heterogeneous microbial communities living in tight association with surfaces. In these communities, bacteria are in continuous competition for resources and space, which could affect the spread of antibiotic-resistant microbial strains. Resistance is typically measured in planktonic cultures using the minimum inhibitory concentration (MIC), which is the lowest concentration of antimicrobial agent that will inhibit microorganism growth [53]. For example, Gram-negative are intrinsically more resistant to antibiotics such as vancomycin (VAN) than Gram-positive cells due to the relative impermeability of the Gram-negative outer membrane. Beyond classic resistance mechanisms, bacteria may also exhibit a multifactorial “tolerance” as the ability to survive at transient exposure to high antibiotic concentration. A measure of tolerance is the minimum bactericidal concentration (MBC), which is the lowest concentration of a bactericidal antimicrobial that will kill ≥99.9% of cells in culture [53].

Currently, the most common antibiotic-resistant microorganisms include *Enterococcus faecium*, *Staphylococcus aureus*, *Klebsiella pneumonia*, *Acinetobacter baumannii*, *Pseudomonas aeruginosa*, and *Enterobacter* species, also known as “ESKAPE” microorganisms [54]. oDAIs include methicillin-resistant *S. aureus* (MRSA), vancomycin-resistant *S. aureus* (VRSA), multidrug-resistant *Acinetobacter*, extended-spectrum β-lactamase producing *Enterobacteriaceae*, and multi-drug-resistant *P. aeruginosa* [54].

Antibiotic biofilm resistance may express through several protective mechanisms, involving a high number of resistant microbial strains, EPS matrix low-permeability toward antibiotics, accumulation of antibiotic-degrading enzymes in the matrix, antibiotic expulsion via efflux pumps, cell heterogeneity in metabolism and growth rate, quorum sensing, persistent cells, genetic adaptation and mutations, adaptive stress response, interactions between different types of bacteria in polymicrobial biofilms [54,55]. For instance, *P. aeruginosa* biofilm produces Psl, an exopolysaccharide made up of repeating pentasaccharide subunits of D-glucose, D-mannose, and L-rhamnose, providing tolerance to aminoglycoside antibiotics (gentamicin, polymyxin B, and ciprofloxacin) at the early stages of biofilm development. Oxacillin, cefotaxime, and VAN cross the biofilm of *S. aureus* and *S. epidermidis* to a limited extent. On the other side, antibiotic-degradating enzymes (i.e., β-lactamases) may also accumulate in the biofilm matrix, hampering cellular target reaching. For example, ampicillin resistance in *K. pneumonia*, penicillin resistance in *S. aureus* [56], imipenem, and ceftazidime resistance in *P. aeruginosa PAO1-J32* were the consequence of antibiotic hydrolysis catalyzed by β-lactamases. Contrary to penicillin resistance, methicillin resistance is a result of drug target modification.

In recent years, it has been proved that quorum-sensing systems might promote antibiotic-resistant-strains survival in competing multispecies communities by modifying their gene expression depending on the microenvironment conditions. For instance, the *agr* system activation in *S. aureus* has been associated with resistance to cephalosporins, VAN, daptomycin, linezolid, rifampicin, and fusidic acid [56].

## 5. Common Standard Treatments Used in the MDIs Management

Cardiac and orthopaedic indwelling device implantation is currently a successful surgical procedure aimed at providing pain relief, restoring organ function, and significantly improving patients’ QoL. However, IE, OM, and PJI are adverse complications of cardiac and orthopaedic surgery, with a high risk of morbidity, mortality, and a substantial healthcare burden. To minimize the overall incidence of infection, several strategies have been proposed, for example, host risk factors identification, patients’ health modification, proper wound care, and operative room environment optimization [20,57]. In this regard, an effective infection risk preventive strategy is represented by the pre-operative “7 S care bundle” approach [58,59].

Guidelines related to antibiotics use aimed at mitigating sepsis related to cardiac and bone device implantations have been widely revised [60]. Broad-spectrum antibiotics, including cefazolin, VAN, clindamicin, rifampicin, amoxicillin, and clavulanic acid, are standard clinical practices in cardiac and orthopaedic procedures [60], ensuring a coverage toward *S. aureus*, MRSA, Gram-negative bacilli, and beta-hemolytic Streptococci. Systemic antibiotics prophylaxis is routinely recommended starting at least 1 h before surgery and up to 48 h after implantation. Debridement and implant retention are conservative therapeutic strategies employed for early oDAIs. This approach has proved a highly variable success rate (0–89%), with the greatest impact for early-diagnosed infections (within 1 month), in healthy patients with mildly aggressive bacteria. This success rate goes down when dealing with very virulent microorganisms, such as MRSA. Two-stage implant exchange, one-stage implant exchange, permanent resection arthroplasty, and amputation are conservative strategies employed in late-diagnosed infections. For instance, two-stage exchange in PIJ surgery consists of debridement of necrotic tissue, infected implant resection followed by a temporary antibiotic-impregnated cement spacer placement, and delayed re-implantation of a new prosthesis in a separate surgery after the infection has been eradicated [20]. Long-term systemic antibiotic therapy is used when implant removal is not possible.

However, an insufficient antibiotic concentration at the infective site, as well as antibiotic-resistant bacteria growing and the risk of systemic toxicity, are some of the main limitations of systemic prophylaxis. To overcome these issues, an initial localized antibiotic burst release to combat bacteria encountered during surgery followed by a sustained release profile to eradicate the hematogenous spread of bacteria at the surgical site for a prolonged period might represent a successful strategy. For instance, antibiotic-loaded bone cement or fillers are widely accepted as local prophylaxis in total hip or knee arthroplasty practice.

Several studies have focused on the limitation of antibiotic prophylaxis on IE incidence related to cardiac devices. In France, the administration of antibiotics oral therapy is limited to high-risk cases since the early 2000s, although no significant change in the incidence of oral streptococcal infection was reported [39]. Meanwhile, the American College of Cardiology/America Heart Association (ACC/AHA) limited antibiotic prophylaxis to the presence of valve prosthesis biomaterials, coronary heart disease, and heart transplants in 2007, arguing no increase in IE incidence [39]. On the contrary, a decrease in *Streptococcus viridans* infection rate has been found from 3.6% per 100,000 person-years (1999–2002) to 1.5% per 100,000 person-years [39]. In the U.K., antibiotic prophylaxis associated with cardiac implants has been drastically reduced to 1/5 of the doses according to the U.K.’s National Institute of Health and Care Excellence Guidelines in 2008.

Unlike orthopaedic devices, biomaterials commonly used in cardiological devices lack systems to counteract the harmful effects of biofilm formation. Therefore, antibiotic therapy is the only possible prevention route. Steffen et al. strongly supported the use of allografts in extensive cardiac structural infections, both in native and prosthetic valvular disease [61]. The authors have shown that cryopreserved human allografts (CHAs) have antibacterial activity despite long-term conservation for 5 years. Antibiotic combinations (gentamicin, piperacillin, VAN, metronidazole, amphotericin B, flucloxacillin, meropenem, tobramycin, and colistin) applied during allogeneic tissue processing have a significant influence on their infection resistance [61]. Kuehn et al. revealed that usage of antibiotic after thawing cryopreserved homograft led to a significant decline in the recurrence of infections [62], while this process is not highlighted in the conventional prosthesis or Dacron graft, although the risk of vascular graft infection is decreased by pre-treating the prosthesis with antibiotics [63]. Indeed, the antibiotic/fibrin compound showed a favourable effect of delayed antibiotics releasing in the early prevention of infection relapse [63]. Furthermore, new titrations of more effective concentrations of β-lactam antibiotics may enhance this action by providing additional immunity in the recurrence of the infected field [63]. The favourable response of homograft to antibiotic usage is already documented in the pivotal series where the cryopreserved biological derivates were successfully processed medically (from 21% to 25%) [64].

Finally, allogenic and autologous tissues have demonstrated favourable responses to antibiotic treatment (effective in 21% to 25% of cases) [64,65,66]. Several reports recorded a low rate (0.2%) of infection relapse at 30 days and 5.5% of late infection with a median time of 5 years (4 months to 16 years) post-allograft implantation in homograft recipients with aortic IE. Two studies with a follow-up over 20 years showed excellent results post-operatively using aortic homografts, with a low incidence of reoperation for relapsing infections (2.2%) [64,65]. Nappi et al. reported the absence of recurrent infection with the use of living pulmonary autograft for replacement of diseased aortic valve at 23 years follow-up [66].

## 6. Passive and Active Antibiofilm Treatments

There is no doubt that in cardiac and orthopaedic indwelling devices design, the focus should be on identifying new approaches to simultaneously stimulate host tissue integration while preventing bacteraemia and counteracting microbial adhesion and colonization of materials’ surface [20]. Several strategies have been pursued in the last few years, identified as passive and active surface modifications.

Passive antibiofilm strategies, also known as antifouling, aim at hampering or reducing bacterial adhesion to implants through surface chemistry and/or structure modifications without using any pharmacologically active compounds. Although promising, passive coatings revealed a limited efficacy in vivo and clinical practice. On the contrary, active antibiofilm strategies aim at killing bacteria via direct contact or by releasing pre-loaded antimicrobials or other compounds, able to hamper biofilm formation or to increase susceptibility toward antimicrobials peri-operative antibacterial local carriers or coatings, which are applied during surgery, immediately before implant placing, represent a successful strategy in orthopaedic [13]. Currently, only four technologies have reported clinical outcomes [67]: silver and iodine coatings, gentamicin poly(D, L-lactide) (PLLA) coating, and a fast-resorbable hydrogel coating composed of covalently linked hyaluronan and PLLA (Defensive Antibacterial Coating (DAC) Novagenit Srl, Mezzolombardo, Italy). All antifouling and antimicrobial biomaterial-based surface treatments reviewed in the present work with corresponding physic-chemical, in vitro, and in vivo results are listed in Table 3.

### 6.1. Passive Antifouling Strategies in cDAIs and oDAIs

Passive antifouling strategies rely on modifying nano-topography, roughness, electrostatic charge, and hydrophobicity of implants’ surface. These modifications confer adhesion-resistant or bacteria-repellent features to the material, targeting the phase (1) of biofilm formation (see Figure 1).

**Figure 2:**
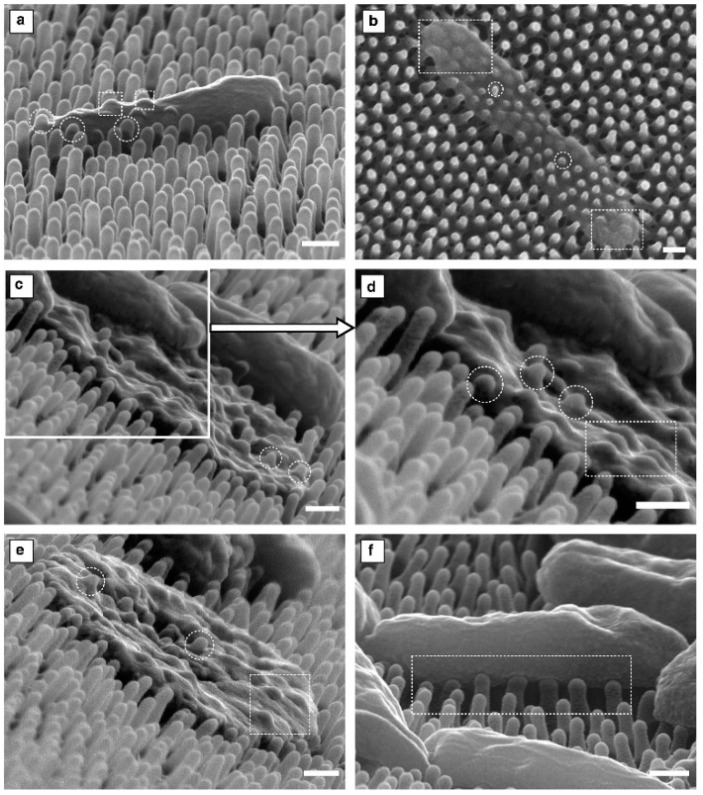
SEM micrographs of Gram-negative *P. aeruginosa* adhered to the nanopattern of the cicada wing surface differing in nanopillar tips deformation could induce: (**a**) a slight penetration in the bacterial envelope without affecting its shape and turgor; (**b**–**e**) an irreversible penetration into bacterial envelope resulting in total loss of turgor; (**f**) bacterial body perturbation. Circular and rectangular outlines are used to highlight penetration and perturbation, respectively (Scale bars 200 nm). Reprinted from *The Lancet*, [68], copyright 2021, with permission from Elsevier.

Surface nano-patterning was inspired by several examples of natural antifouling surfaces, such as lotus leaves, cicada, and dragonfly wings. In light of this, precise surfaces have been patterned with nanostructures (i.e., ordered stripes, pits, pillars, or squares), differing in size, height, width, depth, and spacing. These nanopatterned surfaces have proved their antifouling and/or killing effectiveness versus *Streptococcus*/*Staphylococcus spp.* and *P. aeruginosa* by inducing physical deformation. In this regard, Velic and co-workers demonstrated, through a biophysical modelling approach, the mechanics of *P. aeruginosa* interaction and death on nanopatterned *P. claripennis* cicada wing. Their results suggested that higher depth pillar tips created a critical site at the pillar apex, resulting in penetration through the bacterial envelope with a following partial (Figure 2a) or total (Figure 2b–e) loss of turgor; while lower depth pillar tips resulted only in a perturbation of the bacterial body (Figure 2f) [68].

Microbial anchoring may be precluded by changing the wettability of implant surfaces by using zwitterionic polymers or (super-)hydrophobic chemistry. For instance, UV-C irradiation increased “spontaneous” wettability on Ti6Al4V wire surfaces implanted into the medullar channel of rat femurs, inhibiting *S. aures* adhesion (70.48%) inoculated into the femurs canal, while ensuring a successful implants osteointegration up to nearly 100% at week 4 of healing [69]. Ti6Al4V surface covalent grafting with zwitterionic polymer brushes poly(sulfobetaine methacrylate) (pSBM) significantly reduced *S. aureus* colonization in a mouse femoral intramedullary canal infection model, also combined with a single-dose VAN injection [70]. A pulsed electrodeposition of polyethylene glycol (PEG) coating on Ti surfaces drastically reduced *S. aureus* and *E. coli* attachment (90%) while keeping human fibroblast adhesion [71].

**Figure 3:**
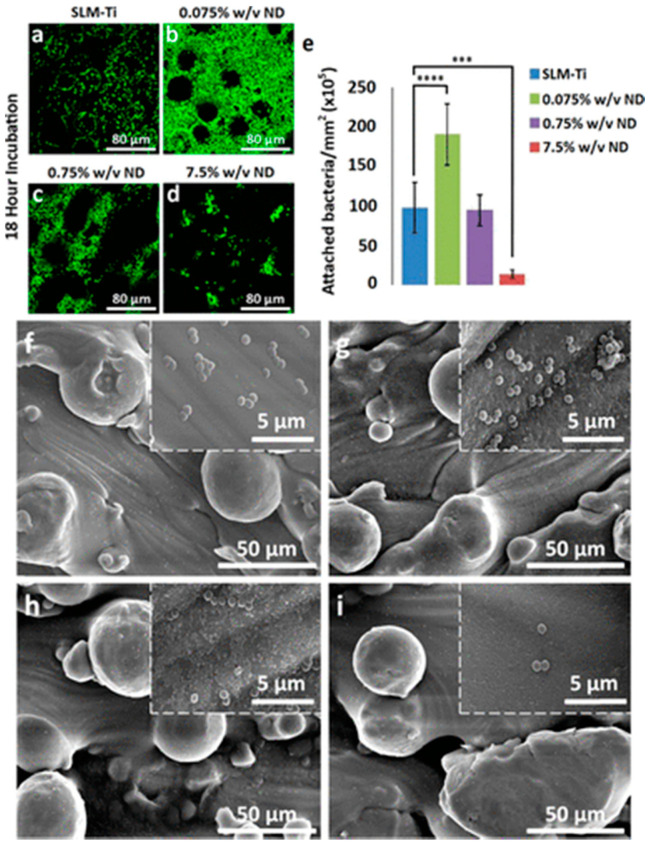
*S. aureus* adhesion and growth on nanodiamond (ND)-coated selective laser melted titanium (ND) substrata (ND-SLM-Ti). Live/Dead staining of *S. aureus* growth on (**a**) uncoated SLM-Ti, (**b**) 0.0075% *w*/*v* ND, (**c**) 0.75% *w*/*v* and (**d**) 7.5% *w*/*v* ND-coated SLM-Ti substrates after 18 h of incubation. (**e**) *S. aureus* density on the uncoated SLM-Ti and SLM-Ti coated with 0.075–0.75–7.5% *w*/*v* ND quantified from Live/Dead fluorescent images after 18 h of incubation. The *S. aureus* density is expressed as average cell number per mm^2^ and indicated as mean ± standard deviation, n = 3. *p* < 0.01. SEM micrographs of *S. aureus* adhesion on (**f**) uncoated SLM-Ti, (**g**) 0.0075% *w*/*v* ND, (**h**) 0.75% *w*/*v* and (**i**) 7.5% *w*/*v* ND-coated SLM-Ti substrates after 18 h of incubation (Mag. 1000×, scale bar 50 µm; insert Mag. 30,000×, scale bar 5 µm). Reprinted and adapted with permission from [73]. Copyright 2021, American Chemical Society.

Hierarchical surface nano-topography and chemistry finely combined ensure outstanding anti-repellent properties based on (super-)hydrophobic features. Quercitrin-functionalized via wet chemistry of porous structure of Ti6Al4V orthopaedic implants allowed promoting osseointegration and preventing infection by decreasing bacterial adhesion by 75% [72]. Nanodiamond coating (at 0.075, 0.75, and 7.5% *w*/*v*) onto additively manufactured selective laser melted Ti substrates synergistically promoted osseointegration and inhibit *S. aureus* adhesion by 88%, as shown in Figure 3 [73].

### 6.2. Active Antimicrobial Strategies in cDAIs and oDAIs

Active antimicrobial strategies involve the use of inherent antimicrobial thin coating intended for killing adherent microorganisms via direct contact or antimicrobial agents’ release. Among active antimicrobial agents used there are metals (i.e., silver (Ag), cerium oxide (CeO_2_), titanium dioxide (TiO_2_), etc.), non-metal elements (i.e., iodine, selenium (Se)), antimicrobial enzyme (i.e., lysozyme), antiseptic agents (i.e., chlorhexidine, chloroxylenol, poly-hexamethylenebiguanide), organic substances (i.e., polyethylenimine, porphyrin, quaternary ammonium compounds (QACs), chitosan (CS)), antibiotics (i.e., aminoglycoside, glycopeptides, penicillins, quinolones, rifamycin, tetracyclines) and their combinations.

Antimicrobial contact-killing surfaces stem from specific antibacterial agents anchored via irreversible covalent bonding or physical absorption. Antimicrobial efficacy of metal and non-metal-based compounds takes place by damaging cell membrane, producing reactive oxygen species, blocking transmembrane transport processes, damaging DNA, and inhibiting enzymatic activity. Ag-based NPs are the most widely used biochemically active agents against a broad spectrum of bacteria, namely *P. aeruginosa*, *S. aureus*/*epidermidis*, and *MRSA*, *E. coli*, and *K. pneumonia* [74]. However, the use of silver-coated implants in orthopaedic and cardiac clinics has long been debated among the scientific community due to their toxicity. Although clinical safety and efficacy have been demonstrated [74], routine use of silver-coated implants remains limited. This issue forced researchers to look for alternative antimicrobial agents. Recently, CeO_2_ NPs proved to own a relatively lower (or even no) toxicity versus mammalian cells [75] than Ag NPs, and do not need to be externally activated as TiO_2_ NPs. CeO_2_ NPs induce oxidative stress through their reversible and mixed valance states (Ce^4+^↔Ce^3+^). Ti surfaces modified cube-CeO_2,_ and octa-CeO_2_ NPs strongly inhibited early Gram-positive adhesion (i.e., *S. sanguinis*) but not of Gram-negative (i.e., *F. nucleatum*) due to probable differences in the extra outer membrane surrounding Gram-negative’s peptidoglycan layer [76]. In another work, Ti implant surface functionalized with Ce-doped HA/Coll coating displayed powerful antibacterial properties by killing 92.61% *E. coli* and 73.59% *S. aureus* after 24 h of incubation [77]. Lately, non-metal elements such as pure or capped Se NPs proved their strong capability of inhibiting MRSA and MRSE biofilm formation on Se NPs-coated Ti implants in rat femurs in vivo model and reducing the number of viable bacteria in the surrounding [78].

**Figure 4:**
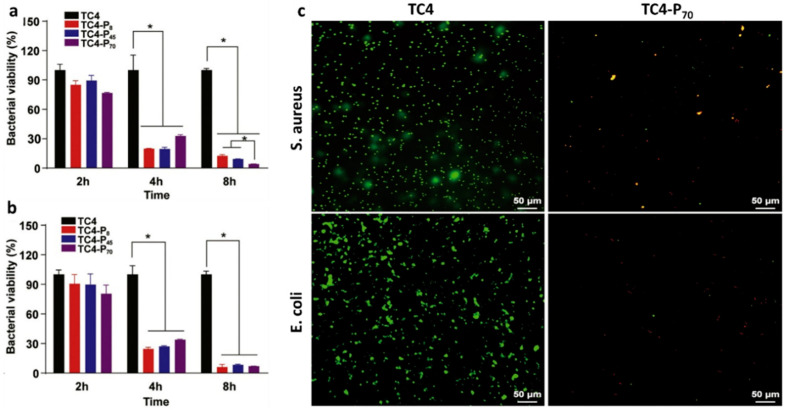
Antibacterial efficacy of the Ti alloy substrates coated with different QAC polymers against (**a**) *S. aureus* and (**b**) *E. coli*. Data are shown as mean ± SEM (n = 3). Statistical significance was determined by two-way ANOVA multiple comparison tests. Pairwise comparisons are statistically significant as denoted as *. (**c**) Confocal laser-scanning microscopy images of *S. aureus* and *E. coli* after 8 h of incubation on neat and Ti alloy and TC4-P70 substrates, respectively. Reprinted from *The Lancet*, [83], copyright 2021, with permission from Elsevier.

Polycationic polymers, such as CS and QACs, have caught the attention of many researchers owing to their inherent pathogens’ contact-killing capability. CS has been widely used for its antibacterial features, even if the precise mechanism is yet to be fully understood. Two main hypotheses have been suggested: (i) positively charged CS might interact with negatively charged microbial cell surfaces, altering membrane permeability, inhibiting DNA replication and RNA synthesis and leaking of cellular contents; (ii) CS could act as a chelating agent, binding trace metal elements and causing toxin production with resultant microbial growth inhibition [79]. A polylactide-co-glycolide hydroxyapatite 3D-printed scaffold grafted with a quaternized chitosan-(PLGA/HA/HACC) exhibited outstanding antimicrobial efficacy versus MSSA and osteoconductive properties in repairing infected cortical and cancellous bone defects in rat and rabbit in vivo models [80]. QACs are cationic surfactants, with long alkyl groups and quaternary ammonium groups, well known as disinfection agents. QAC antimicrobials are long-lasting contact-based antibacterial agents, effective against both Gram-positive and Gram-negative, including multidrug-resistant strains, fungi, and certain classes of viruses [81]. Their antibacterial activity lies on the positively charged quaternary amine N^+^, which changes the ionic balance of the bacterial cell (i.e., sodium (Na^+^), potassium (K^+^), Mg^2+^, Ca^2+^), disrupting the membrane [82]. Recently, a novel phosphonate/quaternary amine block polymer (pDEMMP_15_-b-pTMAEMA_70_) coating of Ti alloy plates (TC4-P_70_) exhibited an antibacterial rate of 95.8% of *S. aureus* and 92.9% of *E. coli* cells attached, as shown in Figure 4 [83]. Meanwhile, Bouloussa et al. assessed an N^+^/N ratio-dependent killing activity of quaternized polyvinylpyridine-grafted (Q-PVP) Ti plates against MRSA [84].

Antibiotics, alone or in combination with other antimicrobial compounds, have been proposed as local release strategies by embedding or immobilizing onto implant coatings [13]. Several coating techniques involving biocompatible synthetic/natural polymers or ceramics nanostructures and antibiotics have been investigated [85]. Among naturally and synthetically polymers commonly used there are CS, Coll, PDLLA, PLA, PLGA, poly(β-amino esters)/poly(acrylic acid), and poly(ethylene glycol)-poly(lactic-co-caprolactone) [85].

Gentamicin-based coatings are the most studied owing to the well-established use of this antibiotic in the clinic for MSSA bone-related infections treatments [86,87,88,89,90]. Vancomycin, commonly used to treat MRSA infections, has also been incorporated into implant coatings. Additional antibiotics, such as fosfomycin, rifampin, ciprofloxacin, levofloxacin (Levo), and cefuroxime, have also been incorporated into implant coatings [91,92,93]. The release mechanism of the pre-loaded antibiotics may occur by degradation of the surface coating, hydrolysis of the covalent bonds, or diffusion [94]. Ideally, to win the fight against microbial infections associated with implants, a biphasic antibiotic-release system that enables a boost early-stage release to immediately eradicate any bacteria, followed by a sustained antibiotic release above the MIC value to kill any remaining bacteria, might be a successful strategy [93].

**Figure 5:**
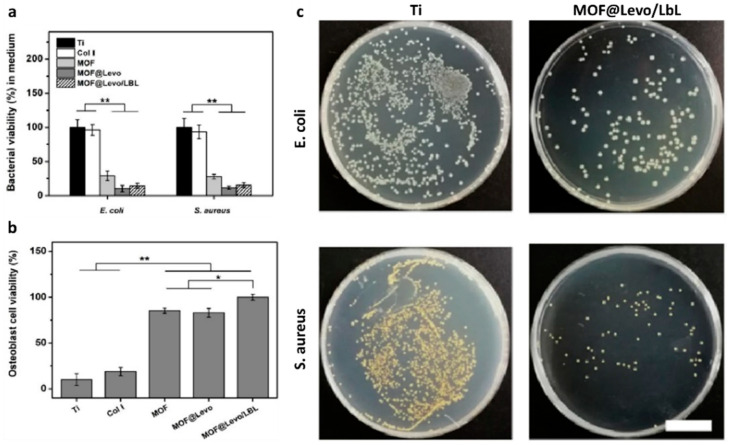
(**a**) *E. coli* and *S. aureus* viability in culture medium after incubation with Ti, Col I, MOF, MOF@Levo and MOF@Levo/LBL surfaces; (**b**) osteoblasts viability on Ti, Col I, MOF, MOF@Levo and MOF@Levo/LBL surfaces in a co-culture model (n = 6, * *p* < 0.05, ** *p* < 0.01); (**c**) pictures of re-cultivated *E. coli* and *S. aureus* colonies on LB agar plate after incubation with Ti and MOF@Levo/LBL surfaces (scale bar 2 cm). Reprinted from *The Lancet*, [92], copyright 2021, with permission from Elsevier.

VAN-loaded niosomes coating stainless steel-based bone plates have exhibited a high antibiotic loading capacity, along with a prolonged release up to two weeks, which was above (125 μg/mL) the MIC value of VAN against *S. aureus* (8 μg/mL) [95]. In another work, covalent grafting of VAN-bearing polymer brushes on Ti6Al4V implant surface (Ti-pVAN) has produced antimicrobial coatings able to significantly suppress *S. aureus* colonization in vitro and in the mouse intramedullary canal (≈20-fold) [96]. VAN, ciprofloxacin, and cefuroxime loaded CS sponges represent a suitable carrier matrix for antibiotic sustained release up to 30 days in oDAIs [91]. Tao and co-workers proposed Levo-loaded zeolitic imidazolate framework-8 NPs (MOF-8@Levo) electrodeposited on Ti substrates and spin-coated with (Gel/CS)_5_ multilayers (identified as MOF@Levo/LBL) [92]. MOF@Levo/LBL Ti-based implant exhibited simultaneously significant antibacterial property against *E. coli* (88.5%, see Figure 5)) and *S. aureus* (86.4%, see Figure 5) through a simultaneous release of Levo and Zn^2+^, and enhance in vitro osteoblastic behavior (see Figure 5), upregulating early-stage osteogenic differentiation markers (Col I and Runx2). Furthermore, MOF@Levo/LBL Ti-based implant also had a positive osseointegration effect and superior antibacterial activity in a femur-infected rat in vivo model [92]. Recently, Ferguson et al. proposed a novel surgical approach based on gentamicin-eluting synthetic bone graft substitute (GEN-BGS) (60% fast resorbing calcium sulfate and 40% HA) in the clinical treatment of chronic osteomyelitis infections associated with fractures and implants [89]. Chronic osteomyelitis infections were eradicated in 95.7% of patients treated with a single procedure. GEN-BGS efficacy in managing dead-space in surgically treated chronic osteomyelitis, with a low infection recurrence rate (4.3%) and suitable mean bone void-filling (73.8%), was demonstrated [89].

Finally, additive manufacturing technologies have also been established as a distinctive approach for customized biomaterials engineering, for either bulk or surface properties, to improve implant outcomes [97]. Additive manufacturing of antimicrobial materials is a small but rapidly growing field [98]. In this regard, topological modifications by additive manufacturing have been observed in ossicular prostheses produced by Milazzo et al., a real niche application, demonstrating to improve hearing recovery while facilitating the implantation [99,100,101,102], and which could also be easily integrated for reaching antifouling features.

## 7. Promising Antimicrobial Compounds for the Treatment of Cardiac and Orthopaedic Device-Associated Infections

Antimicrobial peptides (AMPs) represent a promising alternative to conventional antibiotics to reduce the incidence of MDIs [103]. AMPs are small-sized (12–50 amino acids) amphipathic cationic defense molecules widely diffused in nature, which can be found in four structural conformations (α-helix, β-sheet, extended helix, and loops) [104]. Cathelicidins (LL-37), human β-defensins 3 (HBD-3), magainins, and temporins are the most prevalent AMPs. They own anti-infective and immunomodulatory activity toward a broad spectrum of Gram-positive and Gram-negative bacteria, including *MRSA*, and quinolone-resistant *Enterobacteriaceae*, fungi, and viruses [105]. Their antimicrobial activity lies in the positive charges of arginine and lysine residues [106] and hydrophobicity [106], through which AMPs attach to the bacteria membrane. Once in contact with the bacterial cell membrane, AMPs create pores on the bacterial cell membrane, disrupting the osmotic balance and causing cell lysis, or translocate through the bacterial cell membrane, triggering intracellular pathways of cell death [106]. Due to this membrane destabilizing mechanism, the AMPs are less prone to pathogen resistance development than antibiotics. AMPs can be directly incorporated or immobilized into coatings intended for implant surface through layer-by-layer assembly, adsorption, or covalent bonding [107].

Ti alloy surface functionalized with a nano-HA coating pre-loaded with HBD-3 antimicrobial peptide and bone morphogenetic protein-2 (BMP-2) prevented *E. coli* and *S. aureus* growth for 7 days while promoted adherence and proliferation and osteogenic differentiation of human bone marrow stem cells (hBMSCs) in 7 days [108]. LL-37 peptide-loaded nanopore structures onto Ti-based surface exhibits excellent bactericidal properties toward *S. aureus* and MRSA and bone-promoting capabilities in vitro and in non-infected and *infected* in vivo models [109]. α-helical cathelicidin-derived peptide BMAP27(1–18) grafted Ti disks considerably reduced *S. epidermidis* adhesion upon 2 h, and induced morphological alterations, exerting a rapid contact-killing effect [110]. Recently, Ti surfaces functionalized with a fusion peptide (FP), containing HHC36 antimicrobial and QK angiogenic peptides, exhibited over 96.8% in vitro antimicrobial activity against *S. aureus*, *E. coli*, *P. aeruginosa* and MRSA, while upregulating expressions of angiogenesis-related genes/proteins (VEGF and VEGFR-2) of human umbilical vein endothelial cell (HUVECs) and osteogenesis-related genes/proteins (ALP, COL-1, RUNX-2, OPN, and OCN) of hBMSCs [111]. In vivo findings demonstrated that this FP-engineered implant simultaneously inhibited acute bacterial infection and strongly promoted vascularization and osseointegration after 60 days’ implantation [111].

**Figure 6:**
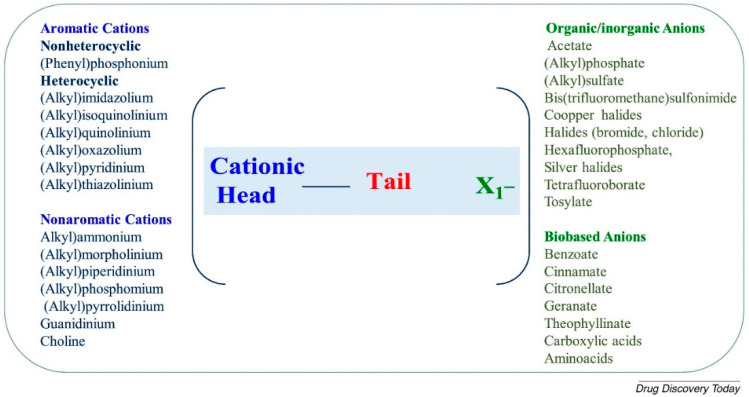
List of the main commonly used cations and anions of ionic liquids. Reprinted from *The Lancet*, [116] Copyright 2021, with permission from Elsevier.

Ionic liquids (ILs) appeared for the first time in the 1970s in the literature, identified as “molten salts”. Only in the mid-1990s, ILs reached popularity as a suitable replacement for volatile organic solvents in Green Chemistry applications [112]. Later, antibacterial activity for monophoshonium ILs, primarily versus Gram-positive organisms, have been demonstrated, confirming the potential utility of these compounds in medicine [113]. ILs are salt in the liquid state with a melting point below 100 °C. They display structural similarities with surfactants consisting of a cationic core (a charged nitrogen-containing organic head group with a linear alkyl chain) responsible for lowering their melting point and a smaller counter-anion responsible for ILs stability in dispersant solution [114]. This peculiar chemical structure gives them outstanding inherent tuneable nature [115]. The most common IL cationic head groups include aromatic (i.e., imidazolium, pyridinium, quinolinium) or non-aromatic (i.e., ammonium, morpholinium, phosphonium, pyrrolidinium, guanidinium, and choline) moieties (Figure 6). The negatively charged anion groups include inorganic (i.e., Cl^−^, AlCl_4_^−^, PF_6_^−^, PF_4_^−^, BF_4_^−^, NTf_2_^−^, DCA^−^), organic (i.e., H_3_COO^−^, CH_3_SO_3_^−^) or amino acids (i.e., proline, tryptophan, phenylalanine, methionine, and valine) (Figure 6) [116]. The length and the number of alkyl chains in the molecule are the main factors determining the antimicrobial activity of ILs, displaying a broad action spectrum toward both Gram-positive and Gram-negative bacteria, as well as mycobacteria and fungi [115]. Antimicrobial activity was higher for ILs containing from 10 to 16 carbon atoms in the alkyl chain than ILs with 8–14 carbon atoms in the alkoxymethyl group.

The complete antimicrobial mechanism of action for all several ILs has not yet been established. These organic electrolytes mainly interact with the lipid membrane of bacterial cells through their alkyl chain, leading to the formation of ion channels, disrupting the intracellular potential and bacteria death [117]. However, evidence in the literature indicates that not all ILs behave similarly. Furthermore, ILs may represent a valid alternative to overcome the antibiotic resistance issue. The possibility of combining antimicrobial compounds (i.e., antibiotics, metal ions, and so on) to the ILs-anion group allows reducing the MIC and MBC of the antibiotic itself, and hence using smaller doses of antibiotics [117]. In addition, antibiotic-ILs complexes exhibited a synergistic antimicrobial effect, owing to enhanced absorption and tissue distribution, along with a wider antibacterial spectrum [117].

Imidazolium-based ILs are the systems most frequently used in biofilm control. To date, very few studies have demonstrated the antimicrobial efficacy of ILs at the pre-clinical level. Gindri and colleagues proposed a dicationic imidazolium-based ILs with amino acid (Phenylalanine and Methionine, IonL-Phe and IonL-Met, respectively) anions as coatings for titanium dental implant providing in vitro strong antimicrobial and antibiofilm activity against *S. mutans*, *S. sanguinis*, and *S. salivarius*), while keeping compatibility with bone and soft tissue forming cells [118]. Recently, a calcium phosphate-imidazolium IL injectable material, revealing antimicrobial and regeneration features, has been proposed as implants in minimally invasive surgery [119].

Polymerized ionic liquids (PILs) can also self-assembled into polymeric nanoparticles, with highly ordered inner structures [120], showing different morphologies, sizes, and surface charges. PILs gained great interest because of their effect on Gram-positive or Gram-negative fungi and algae [121]. A hybrid zinc-based particle coated by 1-n-butyl-3-methylimidazolium chloride (BMI.Cl) was used as filler in dental adhesive resin, providing antibacterial activity against *S. mutans* without changes in the pulp cells’ viability in dental adhesives [122]. Claus and co-workers performed an inherent antibacterial activity screening of 11 different PILs-based hydrogels, reaching a 70% killing efficacy toward MRSA Xen 30 and *P. aeruginosa* Xen 5 [123].

Although ILs have been extensively investigated as promising antimicrobial compounds alternative to antibiotics, there have been not yet pre-clinical outcomes on their efficacy as coating of orthopaedic and cardiac medical devices.

**Table 3:** Antifouling and antimicrobial surface treatments reviewed in the present work: physic-chemical, *in vitro*, and *in vivo* properties.

Material	Functionalization Treatment	Physico-Chemical Features	*In Vitro* Response	*In Vivo* Response	Ref.
**Passive antifouling strategies**					
Ti6Al4V wires	UV-C irradiation	θ = 12°		*S. aureus* adhesion reduction (70.48%) Osteointegration at 4 weeks	[69]
pSBM-grafted Ti6Al4V pins	Surface-initiated polymerization	θ = 10°	Xen-29 *S. aureus* adhesion and colonization reduction	Xen-29 *S. aureus* colonization suppression in infected mouse femoral canal at 21 days, with a systemic VAN injection at day 7	[70]
PEG-coated Ti disks	Pulsed electrodeposition	θ < 5°	*S. aureus* and *E. coli* adhesion reduction (90%) Human fibroblast adhesion supporting		[71]
Quercitrin-grafted Ti6Al4V implant	Wet chemistry	Pore size 500 µm Porosity 52% E = 5.58 ± 0.3 GPa	*S. epidermidis* adhesion reduction (75%) Osteoinductive properties		[72]
NDs-coated Ti plates	Dip-coating	θ decreased by increases NDs concentration	*S. aureus* adhesion reduction (88%) Human dermal fibroblasts (32%) and Osteoblasts (29%) proliferation at 3 days		[73]
**Active antimicrobial strategies**					
Rod-/Cube-/Octa-CeO_2_-coated Ti disks	Spin coating	θ ≈ 37–38°	Saliva-protein repellent activity Anti-inflammatory effects and ROS-scavenging ability *S. sanguinis* early adhesion and colonization inhibition *P. gingivalis* biofilm formation reduction at 4 days	Anti-inflammatory effect in an *in vivo* rat model	[76]
CeO_2_-doped HA/Coll coated Ti plate	Biomimetic wet chemistry		*E. coli* (92.61%) and *S. aureus* (73.59%) bactericidal effect at 24 h		[77]
Se NPs-coated Ti plates and screws	Surface-induced nucleation-deposition	Se NPs size 30–70 nm		Biofilm formation and associated local contamination inhibition MRSA and MRSE bacteria pre-inoculated in rat femurs implants	[78]
PLGA/HA/HACC scaffold	Covalent grafting	σ_Compr_ ≈ 31.3 ± 0.5 MPa σ_Tensile_ ≈ 21.5 ± 0.6 MPa E ≈ 1.9 ± 0.2 GPa	Antimicrobial and osteoconductive properties	Low bacteria burden (at 8 weeks) and new bone formation (at 4 weeks) in femoral shaft and condyle collected in rats and rabbits *in vivo* animal model	[80]
pDEMMP_15_-b-pTMAEMA_70_-coated TC4 plate	Covalent binding	θ ≈ 39.5 ± 7.3°	*S. aureus* (95.8%) and *E. coli* (92.9%) cells adhesion reduction		[83]
(Q-PVP)-Ti plates	Spin-/Dip-coating		Suitable bactericidal effect against MRSA and biocompatibility toward fibroblast and osteoblast-like cells		[84]
VAN-loaded niosomes coated stainless steel plates	Dip-coating	Niosomes: Size ≈ 340.5 ± 2.95 nm ξ ≈ −45.4 ± 0.77 mV EE ≈ 50.47 ± 3.66% DL ≈ 19 ± 1.77%	VAN MIC ≈ 8 µg/mL vs. *S. aureus* VAN MBC ≈ 125 µg/mL vs. *S. aureus* Long-time bactericidal effects No cytotoxicity vs. fibroblast cells		[95]
Ti-pVAN	Surface-initiated atom transfer radical polymerization		*S. aureus* adhesion and colonization reduction	*S. aureus* adhesion and colonization reduction (∼20-fold) supported by VAN in mouse femurs canals *in vivo* model at 21 days	[96]
MOF@Levo/LBL Ti foils		θ ≈ 27.5 ± 1.9°	Strong antibacterial effect vs. *E. coli* and *S. aureus* Osteoblasts adhesion and proliferation stimulation Early-stage (Runx2, ColI) and late-stage (OPN, OPC) osteogenic differentiation markers up-regulation	Osteointegration effect and antibacterial activity in rat model with *S. aureus*-infection	[92]
GEN-BGS				Infection eradication (95.7%) Infection recurrence rate (4.3%) Bone void-filling (73.8%)	[89]
**Innovative anti-microbial biomaterials**					
HBD + BMP/HA-Ti	Dip-coating	BMP EE > 74% HBD-3 and BMP-2 synchronized slow, sustained release 40% up to 90% at 10 days	*S. aureus* and *E. coli* adhesion inhibition hBMSCs adhesion, proliferation, and osteogenic differentiation		[108]
LL37- foils and wires	Simplified lyophilization method	θ ≈ 29.5 ± 3.9° LL37 sustained release within 7 d	*S. aureus* and MRSA proliferation inhibition Osteoinductive capabilities	Osteointegration capacities at 8 weeks in the femur of rat model *S.aureus* pre-infected	[109]
BMAP27(1–18)-coated Ti disks	Covalent binding	θ ≈ 68.3 ± 0.9°	*S. epidermidis* adhesion reduction No cytotoxic effects on osteoblast-like cells		[110]
QK/AMP-coated Ti implant	Cu(I)-catalyzed azide-alkyne cycloaddition		*S. aureus*, *E. coli*, *P. aeruginosa,* and MRSA bactericidal effect (96.8%) HUVECs and hBMSCs adhesion, proliferation, angiogenic markers (VEGF and VEGFR-2), and osteogenic markers (ALP, ColI, RUNX-2, OPN, and OCN) up-regulation	Acute infection inhibition (99.63%), strong vascularization, and osseointegration promotion after 60 days’ implantation in rabbit bone (non-) infected with *S. aureus*	[111]
BMI.ZnCl_3_-coated particles fillers for dental adhesive resin		Size up to 2 μm	*S. mutants* biofilm formation reduction No cytotoxic effects on dental pulp cells		[122]
PILs-based hydrogels			Inherent antibacterial effect (>70%) vs. Gram-negative/-positive bacteria Bactericidal effect (≥68.8%) vs. MRSA		[123]

Abbreviations: NDs = nanodiamonds; θ = contact angle (°); σ_compr_ = compressive strength (MPa); σ_tensile_ = tensile strength (MPa); E = Young modulus (GPa); ξ = z-potential (mV); EE = encapsulation efficiency (%); DL = drug loading (%).

## 8. Conclusions

Multidrug-resistant strains of microorganisms (e.g., *Staphylococci* spp., *Streptococci* spp.) have significant morbidity and mortality implications on cardiac and orthopaedic medical devices in the clinical setting. Ongoing research of new resistant/preventive materials is needed to supplement the currently available therapies. The novel therapies such as antimicrobial peptides and ion liquids offer some promise but need further confirmation to ensure results from studies are translated to clinical practice.

## Figures and Tables

**Table 1:** Summary of the main cardiac and orthopaedic device-associated infections and microbial strains involved.

MDIs	Implanted Devices	Microbial Strains Involved	Ref.
**Cardiac**			
IE	Mechanical heart valves Ventricular shunts Cardiac electronic devices Endovascular stent	*Staphylococci* spp. (70%), *Streptococci* spp. (20%), *Enterococci* spp. (10%), HACEK bacteria group (3%)	[17,18,19]
**Orthopaedic**			
OM	Joint replacement	*Staphylococci* (10–40%), *CoNS* (20–40%), *Enterococci* (3–7%), *Pseudomonas* (≈6%), *E. coli* (<3%)	[17,18,19,20,21]
PIJ			
PI	Dental	*P. gingivalis*, *A. actinomycetemcomitans*, *B. forsythus*, *T. denticola*, *P. nigrescens*, *P. micros*, *F. nucleatum* (30–40%)	[22]
OTM	Cochlear	*S. aureus*, *S. pneumoniae*, *H. influenzae*, *M. catarrhalis* (1–12%)	[23]

## Data Availability

Not applicable.

## References

[1] Afari M.E., Syed W., Tsao L. (2018). Implantable devices for heart failure monitoring and therapy. Heart Fail. Rev..

[2] EMR Global Orthopaedic Implants Market: By Product: Plates and Screws, Nails and Rods, Pins and Wires; By Application; By End Use: Hospitals, Orthopaedic Clinics, Ambulatory Surgical Centres; Regional Analysis; Historical Market and Forecast (2016–2026); Mark. https://www.expertmarketresearch.com/reports/orthopaedic-implants-market.

[3] IMARC Cardiovascular Implants Market: Global Industry Trends, Share, Size, Growth, Opportunity and Forecast 2021–2026. https://www.imarcgroup.com/cardiovascular-implants-market.

[4] Navarro M., Michiardi A., Castaño O., Planell J.A. (2008). Biomaterials in orthopaedics. J. R. Soc. Interface.

[5] Lam M.T., Wu J.C. (2012). Biomaterial applications in cardiovascular tissue repair and regeneration. Expert Rev. Cardiovasc. Ther..

[6] Wilson J. (2018). Metallic biomaterials. Fundamental Biomaterials: Metals.

[7] Seebach E., Kubatzky K.F. (2019). Chronic Implant-Related Bone Infections-Can Immune Modulation be a Therapeutic Strategy?. Front. Immunol..

[8] Bai D., Chen J., Li P., Huang W. (2020). Perspectives on Biomaterial-Associated Infection: Pathogenesis and Current Clinical Demands. Racing for the Surface.

[9] Alexiou K. (2018). Antibiotic prophylaxis in surgery. Surg. Chron..

[10] Ferraris S., Spriano S. (2016). Antibacterial titanium surfaces for medical implants. Mater. Sci. Eng. C.

[11] Hall T.J., Villapún V.M., Addison O., Webber M.A., Lowther M., Louth S.E.T., Mountcastle S.E., Brunet M.Y., Cox S.C. (2020). A call for action to the biomaterial community to tackle antimicrobial resistance. Biomater. Sci..

[12] Milazzo M., Gallone G., Marcello E., Mariniello M.D., Bruschini L., Roy I., Danti S. (2020). Biodegradable polymeric micro/Nano-structures with intrinsic antifouling/antimicrobial properties: Relevance in damaged skin and other biomedical applications. J. Funct. Biomater..

[13] Romanò C.L., Tsuchiya H., Morelli I., Battaglia A.G., Drago L. (2019). Antibacterial coating of implants: Are we missing something?. Bone Jt. Res..

[14] Abt M.C., Pamer E.G. (2014). Commensal bacteria mediated defenses against pathogens. Curr. Opin. Immunol..

[15] Moriarty T.F., Kuehl R., Coenye T., Metsemakers W.J., Morgenstern M., Schwarz E.M., Riool M., Zaat S.A.J., Khana N., Kates S.L. (2016). Orthopaedic device-related infection: Current and future interventions for improved prevention and treatment. EFORT Open Rev..

[16] Giles C., Lamont-Friedrich S.J., Michl T.D., Griesser H.J., Coad B.R. (2018). The importance of fungal pathogens and antifungal coatings in medical device infections. Biotechnol. Adv..

[17] Jamal M., Ahmad W., Andleeb S., Jalil F., Imran M., Nawaz M.A., Hussain T., Ali M., Rafiq M., Kamil M.A. (2018). Bacterial biofilm and associated infections. J. Chin. Med. Assoc..

[18] Muhammad M.H., Idris A.L., Fan X., Guo Y., Yu Y., Jin X., Qiu J., Guan X., Huang T. (2020). Beyond Risk: Bacterial Biofilms and Their Regulating Approaches. Front. Microbiol..

[19] Rajani R., Klein J.L. (2020). Infective endocarditis: A contemporary update. Clin. Med. J. R. Coll. Phys. Lond..

[20] Arciola C.R., Campoccia D., Montanaro L. (2018). Implant infections: Adhesion, biofilm formation and immune evasion. Nat. Rev. Microbiol..

[21] Benito N., Mur I., Ribera A., Soriano A., Rodríguez-Pardo D., Sorlí L., Cobo J., Fernández-Sampedro M., del Toro M., Guío L. (2019). The Different Microbial Etiology of Prosthetic Joint Infections according to Route of Acquisition and Time after Prosthesis Implantation, Including the Role of Multidrug-Resistant Organisms. J. Clin. Med..

[22] Pokrowiecki R., Mielczarek A., Zaręba T., Tyski S. (2017). Oral microbiome and peri-implant diseases: Where are we now?. Ther. Clin. Risk Manag..

[23] Vila P.M., Ghogomu N.T., Odom-John A.R., Hullar T.E., Hirose K. (2017). Infectious complications of pediatric cochlear implants are highly influenced by otitis media. Int. J. Pediatr. Otorhinolaryngol..

[24] Becker K., Heilmann C., Peters G. (2014). Coagulase-negative staphylococci. Clin. Microbiol. Rev..

[25] Nappi F., Spadaccio C., Moon M.R. (2020). A management framework for left sided endocarditis: A narrative review. Ann. Transl. Med..

[26] Mack M.J., Leon M.B., Smith C.R., Miller D.C., Moses J.W., Tuzcu E.M., Webb J.G., Douglas P.S., Anderson W.N., Blackstone E.H. (2015). 5-year outcomes of transcatheter aortic valve replacement or surgical aortic valve replacement for high surgical risk patients with aortic stenosis (PARTNER 1): A randomised controlled trial. Lancet.

[27] Kapadia S.R., Leon M.B., Makkar R.R., Tuzcu E.M., Svensson L.G., Kodali S., Webb J.G., Mack M.J., Douglas P.S., Thourani V.H. (2015). 5-year outcomes of transcatheter aortic valve replacement compared with standard treatment for patients with inoperable aortic stenosis (PARTNER 1): A randomised controlled trial. Lancet.

[28] Regueiro A., Linke A., Latib A., Ihlemann N., Urena M., Walther T., Husser O., Herrmann H.C., Nombela-Franco L., Cheema A.N. (2016). Association between transcatheter aortic valve replacement and subsequent infective endocarditis and in-hospital death. JAMA J. Am. Med. Assoc..

[29] Makkar R.R., Thourani V.H., Mack M.J., Kodali S.K., Kapadia S., Webb J.G., Yoon S.-H., Trento A., Svensson L.G., Herrmann H.C. (2020). Five-Year Outcomes of Transcatheter or Surgical Aortic-Valve Replacement. N. Engl. J. Med..

[30] Mack M.J., Leon M.B., Thourani V.H., Makkar R., Kodali S.K., Russo M., Kapadia S.R., Malaisrie S.C., Cohen D.J., Pibarot P. (2019). Transcatheter Aortic-Valve Replacement with a Balloon-Expandable Valve in Low-Risk Patients. N. Engl. J. Med..

[31] Reardon M.J., Van Mieghem N.M., Popma J.J., Kleiman N.S., Søndergaard L., Mumtaz M., Adams D.H., Deeb G.M., Maini B., Gada H. (2017). Surgical or Transcatheter Aortic-Valve Replacement in Intermediate-Risk Patients. N. Engl. J. Med..

[32] Lanz J., Kim W.K., Walther T., Burgdorf C., Möllmann H., Linke A., Redwood S., Thilo C., Hilker M., Joner M. (2019). Safety and efficacy of a self-expanding versus a balloon-expandable bioprosthesis for transcatheter aortic valve replacement in patients with symptomatic severe aortic stenosis: A randomised non-inferiority trial. Lancet.

[33] Popma J.J., Deeb G.M., Yakubov S.J., Mumtaz M., Gada H., O’Hair D., Bajwa T., Heiser J.C., Merhi W., Kleiman N.S. (2019). Transcatheter Aortic-Valve Replacement with a Self-Expanding Valve in Low-Risk Patients. N. Engl. J. Med..

[34] Makkar R.R., Cheng W., Waksman R., Satler L.F., Chakravarty T., Groh M., Abernethy W., Russo M.J., Heimansohn D., Hermiller J. (2020). Self-expanding intra-annular versus commercially available transcatheter heart valves in high and extreme risk patients with severe aortic stenosis (PORTICO IDE): A randomised, controlled, non-inferiority trial. J. Clean. Prod..

[35] Mihos C.G., Nappi F. (2020). A narrative review of echocardiography in infective endocarditis of the right heart. Ann. Transl. Med..

[36] Nappi F., Spadaccio C., Mihos C., Shaikhrezai K., Acar C., Moon M.R. (2020). The quest for the optimal surgical management of tricuspid valve endocarditis in the current era: A narrative review. Ann. Transl. Med..

[37] Nappi F., Spadaccio C., Mihos C. (2020). Infective endocarditis in the 21st century. Ann. Transl. Med..

[38] Nappi F., Singh S.S.A., Nappi P., Spadaccio C., Nenna A., Gentile F., Chello M. (2020). Heart Valve Endocarditis. Surg. Technol. Int..

[39] Benedetto U., Spadaccio C., Gentile F., Moon M.R., Nappi F. (2020). A narrative review of early surgery versus conventional treatment for infective endocarditis: Do we have an answer?. Ann. Transl. Med..

[40] Mangner N., Woitek F., Haussig S., Schlotter F., Stachel G., Höllriegel R., Wilde J., Lindner A., Holzhey D., Leontyev S. (2016). Incidence, Predictors, and Outcome of Patients Developing Infective Endocarditis Following Transfemoral Transcatheter Aortic Valve Replacement. J. Am. Coll. Cardiol..

[41] Birt M.C., Anderson D.W., Bruce Toby E., Wang J. (2017). Osteomyelitis: Recent advances in pathophysiology and therapeutic strategies. J. Orthop..

[42] (2019). Arthritis Foundation Arthritis by the Numbers. Arthritis Found..

[43] Tande A.J., Patel R. (2014). Prosthetic joint infection. Clin. Microbiol. Rev..

[44] Mombelli A., Müller N., Cionca N. (2012). The epidemiology of peri-implantitis. Clin. Oral Implants Res..

[45] Donlan R.M. (2002). Biofilms: Microbial life on surfaces. Emerg. Infect. Dis..

[46] Berne C., Ellison C.K., Ducret A., Brun Y.V. (2018). Bacterial adhesion at the single-cell level. Nat. Rev. Microbiol..

[47] Flemming H.C., Wingender J. (2010). The biofilm matrix. Nat. Rev. Microbiol..

[48] Colvin K.M., Irie Y., Tart C.S., Urbano R., Whitney J.C., Ryder C., Howell P.L., Wozniak D.J., Parsek M.R. (2012). The Pel and Psl polysaccharides provide Pseudomonas aeruginosa structural redundancy within the biofilm matrix. Environ. Microbiol..

[49] Nguyen H.T.T., Nguyen T.H., Otto M. (2020). The staphylococcal exopolysaccharide PIA—Biosynthesis and role in biofilm formation, colonization, and infection. Comput. Struct. Biotechnol. J..

[50] Subramani R., Jayaprakashvel M. (2019). Bacterial Quorum Sensing: Biofilm Formation, Survival Behaviour and Antibiotic Resistance. Implication of Quorum Sensing and Biofilm Formation in Medicine, Agriculture and Food Industry.

[51] Dhar Y., Han Y. (2020). Current developments in biofilm treatments: Wound and implant infections. Eng. Regen..

[52] Khatoon Z., McTiernan C.D., Suuronen E.J., Mah T.F., Alarcon E.I. (2018). Bacterial biofilm formation on implantable devices and approaches to its treatment and prevention. Heliyon.

[53] Hall C.W., Mah T.F. (2017). Molecular mechanisms of biofilm-based antibiotic resistance and tolerance in pathogenic bacteria. FEMS Microbiol. Rev..

[54] Li B., Webster T.J. (2018). Bacteria antibiotic resistance: New challenges and opportunities for implant-associated orthopedic infections. J. Orthop. Res..

[55] Dincer S., Uslu F.M., Delik A. (2020). Antibiotic resistance in biofilm. Bacterial Biofilms.

[56] Rodis N., Tsapadikou V.K., Potsios C., Xaplanteri P. (2020). Resistance Mechanisms in Bacterial Biofilm Formations: A Review. J. Emerg. Intern. Med..

[57] Boot W., Vogely H.C. (2017). Prevention: Prophylaxis for implant-related infections: Current state of the art. Management of Periprosthetic Joint Infection: A Global Perspective on Diagnosis, Treatment Options, Prevention Strategies and Their Economic Impact.

[58] Ma N., Cameron A., Tivey D., Grae N., Roberts S., Morris A. (2017). Systematic review of a patient care bundle in reducing staphylococcal infections in cardiac and orthopaedic surgery. ANZ J. Surg..

[59] Spencer M.P., Christie J. (2014). A 7 S Bundle Approach To Preventing Surgical Site Infections. Am. J. Infect. Control.

[60] Turner N.A., Sharma-Kuinkel B.K., Maskarinec S.A., Eichenberger E.M., Shah P.P., Carugati M., Holland T.L., Fowler V.G. (2019). Methicillin-resistant Staphylococcus aureus: An overview of basic and clinical research. Nat. Rev. Microbiol..

[61] Steffen V., Marsch G., Burgwitz K., Kuehn C., Teebken O.E. (2016). Resistance to infection of long-term cryopreserved human aortic valve allografts. J. Thorac. Cardiovasc. Surg..

[62] Kuehn C., Graf K., Mashaqi B., Pichlmaier M., Heuer W., Hilfiker A., Stiesch M., Chaberny I.F., Haverich A. (2010). Prevention of early vascular graft infection using regional antibiotic release. J. Surg. Res..

[63] Zander J., Maier B., Zoller M., Döbbeler G., Frey L., Teupser D., Vogeser M. (2016). Effects of biobanking conditions on six antibiotic substances in human serum assessed by a novel evaluation protocol. Clin. Chem. Lab. Med..

[64] Nappi F., Nenna A., Petitti T., Spadaccio C., Gambardella I., Lusini M., Chello M., Acar C. (2018). Long-term outcome of cryopreserved allograft for aortic valve replacement. J. Thorac. Cardiovasc. Surg..

[65] Arabkhani B., Bekkers J.A., Andrinopoulou E.R., Roos-Hesselink J.W., Takkenberg J.J.M., Bogers A.J.J.C. (2016). Allografts in aortic position: Insights from a 27-year, single-center prospective study. J. Thorac. Cardiovasc. Surg..

[66] Nappi F., Spadaccio C., Chello M., Acar C. (2015). The Ross procedure: Underuse or under-comprehension?. J. Thorac. Cardiovasc. Surg..

[67] Alt V. (2017). Antimicrobial coated implants in trauma and orthopaedics—A clinical review and risk-benefit analysis. Injury.

[68] Velic A., Hasan J., Li Z., Yarlagadda P.K.D.V. (2021). Mechanics of Bacterial Interaction and Death on Nanopatterned Surfaces. Biophys. J..

[69] Constantino J.A., Delgado-Rastrollo M., Pacha-Olivenza M.A., González-Martín M.L., Quiles M., Pérez-Giraldo C., Bruque J.M., Gallardo-Moreno A.M. (2017). In vivo bactericidal efficacy of the Ti6Al4V surface after ultraviolet C treatment. J. Orthop. Traumatol..

[70] Zhang B., Skelly J.D., Braun B.M., Ayers D.C., Song J. (2020). Surface-Grafted Zwitterionic Polymers Improve the Efficacy of a Single Antibiotic Injection in Suppressing Staphylococcus aureus Periprosthetic Infections. ACS Appl. Bio Mater..

[71] Buxadera-Palomero J., Albó K., Gil F.J., Mas-Moruno C., Rodríguez D. (2020). Polyethylene glycol pulsed electrodeposition for the development of antifouling coatings on titanium. Coatings.

[72] Llopis-Grimalt M.A., Arbós A., Gil-Mir M., Mosur A., Kulkarni P., Salito A., Ramis J.M., Monjo M. (2020). Multifunctional Properties of Quercitrin-Coated Porous Ti-6Al-4V Implants for Orthopaedic Applications Assessed In Vitro. J. Clin. Med..

[73] Rifai A., Tran N., Reineck P., Elbourne A., Mayes E., Sarker A., Dekiwadia C., Ivanova E.P., Crawford R.J., Ohshima T. (2019). Engineering the Interface: Nanodiamond Coating on 3D-Printed Titanium Promotes Mammalian Cell Growth and Inhibits Staphylococcus aureus Colonization. ACS Appl. Mater. Interfaces.

[74] Kalwar K., Shan D. (2018). Antimicrobial effect of silver nanoparticles (AgNPs) and their mechanism—A mini review. Micro Nano Lett..

[75] Forest V., Leclerc L., Hochepied J.F., Trouvé A., Sarry G., Pourchez J. (2017). Impact of cerium oxide nanoparticles shape on their in vitro cellular toxicity. Toxicol. Vitr..

[76] Li X., Qi M., Sun X., Weir M.D., Tay F.R., Oates T.W., Dong B., Zhou Y., Wang L., Xu H.H.K. (2019). Surface treatments on titanium implants via nanostructured ceria for antibacterial and anti-inflammatory capabilities. Acta Biomater..

[77] Ciobanu G., Harja M. (2019). Cerium-doped hydroxyapatite/collagen coatings on titanium for bone implants. Ceram. Int..

[78] Tran P.A., O’brien-Simpson N., Palmer J.A., Bock N., Reynolds E.C., Webster T.J., Deva A., Morrison W.A., O’connor A.J. (2019). Selenium nanoparticles as anti-infective implant coatings for trauma orthopedics against methicillin-resistant Staphylococcus aureus and epidermidis: In vitro and in vivo assessment. Int. J. Nanomed..

[79] Yilmaz Atay H. (2020). Antibacterial activity of chitosan-based systems. Functional Chitosan: Drug Delivery and Biomedical Applications.

[80] Yang Y., Chu L., Yang S., Zhang H., Qin L., Guillaume O., Eglin D., Richards R.G., Tang T. (2018). Dual-functional 3D-printed composite scaffold for inhibiting bacterial infection and promoting bone regeneration in infected bone defect models. Acta Biomater..

[81] Jiao Y., Niu L., Ma S., Li J., Tay F.R., Chen J. (2017). Quaternary ammonium-based biomedical materials: State-of-the-art, toxicological aspects and antimicrobial resistance. Prog. Polym. Sci..

[82] Jennings M.C., Minbiole K.P.C., Wuest W.M. (2016). Quaternary Ammonium Compounds: An Antimicrobial Mainstay and Platform for Innovation to Address Bacterial Resistance. ACS Infect. Dis..

[83] Liu L., Peng W., Zhang X., Peng J., Liu P., Shen J. (2021). Rational design of phosphonate/quaternary amine block polymer as an high-efficiency antibacterial coating for metallic substrates. J. Mater. Sci. Technol..

[84] Bouloussa H., Saleh-Mghir A., Valotteau C., Cherifi C., Hafsia N., Cohen-Solal M., Court C., Crémieux A.C., Humblot V. (2021). A Graftable Quaternary Ammonium Biocidal Polymer Reduces Biofilm Formation and Ensures Biocompatibility of Medical Devices. Adv. Mater. Interfaces.

[85] Song J., Winkeljann B., Lieleg O. (2020). Biopolymer-Based Coatings: Promising Strategies to Improve the Biocompatibility and Functionality of Materials Used in Biomedical Engineering. Adv. Mater. Interfaces.

[86] Ständert V., Borcherding K., Bormann N., Schmidmaier G., Grunwald I., Wildemann B. (2021). Antibiotic-loaded amphora-shaped pores on a titanium implant surface enhance osteointegration and prevent infections. Bioact. Mater..

[87] Freischmidt H., Armbruster J., Reiter G., Grützner P.A., Helbig L., Guehring T. (2020). Individualized techniques of implant coating with an antibiotic-loaded, hydroxyapatite/calcium sulphate bone graft substitute. Ther. Clin. Risk Manag..

[88] Draghi L., Preda V., Moscatelli M., Santin M., Chiesa R. (2020). Gentamicin-Loaded TiO_2_ Nanotubes as Improved Antimicrobial Surfaces for Orthopedic Implants. Front. Mater..

[89] Ferguson J., Athanasou N., Diefenbeck M., McNally M. (2019). Radiographic and Histological Analysis of a Synthetic Bone Graft Substitute Eluting Gentamicin in the Treatment of Chronic Osteomyelitis. J. Bone Jt. Infect..

[90] McNally M.A., Ferguson J.Y., Lau A.C.K., Diefenbeck M., Scarborough M., Ramsden A.J., Atkins B.L. (2016). Single-stage treatment of chronic osteomyelitis with a new absorbable, gentamicin-loaded, calcium sulphate/hydroxyapatite biocomposite: A prospective series of 100 cases. Bone Jt. J..

[91] Pawar V., Bulbake U., Khan W., Srivastava R. (2019). Chitosan sponges as a sustained release carrier system for the prophylaxis of orthopedic implant-associated infections. Int. J. Biol. Macromol..

[92] Tao B., Zhao W., Lin C., Yuan Z., He Y., Lu L., Chen M., Ding Y., Yang Y., Xia Z. (2020). Surface modification of titanium implants by ZIF-8@Levo/LBL coating for inhibition of bacterial-associated infection and enhancement of in vivo osseointegration. Chem. Eng. J..

[93] Masters E.A., Trombetta R.P., de Mesy Bentley K.L., Boyce B.F., Gill A.L., Gill S.R., Nishitani K., Ishikawa M., Morita Y., Ito H. (2019). Evolving concepts in bone infection: Redefining “biofilm”, “acute vs. chronic osteomyelitis”, “the immune proteome” and “local antibiotic therapy”. Bone Res..

[94] Ahmed W., Zhai Z., Gao C. (2019). Adaptive antibacterial biomaterial surfaces and their applications. Mater. Today Bio.

[95] Dwivedi A., Mazumder A., Nasongkla N. (2018). Layer-by-layer nanocoating of antibacterial niosome on orthopedic implant. Int. J. Pharm..

[96] Zhang B., Braun B.M., Skelly J.D., Ayers D.C., Song J. (2019). Significant Suppression of Staphylococcus aureus Colonization on Intramedullary Ti6Al4V Implants Surface-Grafted with Vancomycin-Bearing Polymer Brushes. ACS Appl. Mater. Interfaces.

[97] Sinha R., Cámara-Torres M., Scopece P., Verga Falzacappa E., Patelli A., Moroni L., Mota C. (2021). A hybrid additive manufacturing platform to create bulk and surface composition gradients on scaffolds for tissue regeneration. Nat. Commun..

[98] Vargas-Alfredo N., Dorronsoro A., Cortajarena A.L., Rodríguez-Hernández J. (2017). Antimicrobial 3D Porous Scaffolds Prepared by Additive Manufacturing and Breath Figures. ACS Appl. Mater. Interfaces.

[99] Milazzo M., Danti S., Inglese F., Jansen van Vuuren G., Gramigna V., Bonsignori G., De Vito A., Bruschini L., Stefanini C., Berrettini S. (2017). Ossicular replacement prostheses from banked bone with ergonomic and functional geometry. J. Biomed. Mater. Res. Part B Appl. Biomater..

[100] Milazzo M., Muyshondt P.G.G., Carstensen J., Dirckx J.J.J., Danti S., Buehler M.J. (2020). De novo topology optimization of total ossicular replacement prostheses. J. Mech. Behav. Biomed. Mater..

[101] Feula M., Milazzo M., Giannone G., Azimi B., Trombi L., Cacopardo L., Moscato S., Lazzeri A., Ahluwalia A., Berrettini S. (2020). Bioartificial Sponges for Auricular Cartilage Engineering. Lecture Notes in Bioengineering.

[102] Milazzo M., Danti S., Inglese F., Berrettini S., Stefanini C. Micro-machining of bovine bone for otologic applications. Proceedings of the Euspen’s 18th International Conference & Exibition.

[103] Wang J., Dou X., Song J., Lyu Y., Zhu X., Xu L., Li W., Shan A. (2019). Antimicrobial peptides: Promising alternatives in the post feeding antibiotic era. Med. Res. Rev..

[104] Pinto I.B., dos Santos Machado L., Meneguetti B.T., Nogueira M.L., Espínola Carvalho C.M., Roel A.R., Franco O.L. (2019). Utilization of antimicrobial peptides, analogues and mimics in creating antimicrobial surfaces and bio-materials. Biochem. Eng. J..

[105] Mahlapuu M., Björn C., Ekblom J. (2020). Antimicrobial peptides as therapeutic agents: Opportunities and challenges. Crit. Rev. Biotechnol..

[106] Raheem N., Straus S.K. (2019). Mechanisms of Action for Antimicrobial Peptides With Antibacterial and Antibiofilm Functions. Front. Microbiol..

[107] Kazemzadeh-Narbat M., Cheng H., Chabok R., Alvarez M.M., de la Fuente-Nunez C., Phillips K.S., Khademhosseini A. (2021). Strategies for antimicrobial peptide coatings on medical devices: A review and regulatory science perspective. Crit. Rev. Biotechnol..

[108] Liu H.W., Wei D.X., Deng J.Z., Zhu J.J., Xu K., Hu W.H., Xiao S.H., Zhou Y.G. (2018). Combined antibacterial and osteogenic in situ effects of a bifunctional titanium alloy with nanoscale hydroxyapatite coating. Artif. Cells Nanomed. Biotechnol..

[109] Shen X., Al-Baadani M.A., He H., Cai L., Wu Z., Yao L., Wu X., Wu S., Chen M., Zhang H. (2019). Antibacterial and osteogenesis performances of LL37-loaded titania nanopores in vitro and in vivo. Int. J. Nanomed..

[110] Boix-Lemonche G., Guillem-Marti J., D’Este F., Manero J.M., Skerlavaj B. (2020). Covalent grafting of titanium with a cathelicidin peptide produces an osteoblast compatible surface with antistaphylococcal activity. Colloids Surf. B Biointerfaces.

[111] Chen J., Hu G., Li T., Chen Y., Gao M., Li Q., Hao L., Jia Y., Wang L., Wang Y. (2021). Fusion peptide engineered “statically-versatile” titanium implant simultaneously enhancing anti-infection, vascularization and osseointegration. Biomaterials.

[112] Pernak J., Rzemieniecki T., Materna K. (2016). Ionic liquids “in a nutshell” (history, properties and development). Chemik.

[113] Brunel F., Lautard C., di Giorgio C., Garzino F., Raimundo J.M., Bolla J.M., Camplo M. (2018). Antibacterial activities of mono-, di- and tri-substituted triphenylamine-based phosphonium ionic liquids. Bioorg. Med. Chem. Lett..

[114] Faisal M., Saeed A. (2020). The role of ionic liquid in medicinal chemistry. Green Approaches in Medicinal Chemistry for Sustainable Drug Design.

[115] Prudêncio C., Vieira M., Van der Auweraer S., Ferraz R. (2020). Recycling old antibiotics with ionic liquids. Antibiotics.

[116] Simões M., Pereira A.R., Simões L.C., Cagide F., Borges F. (2021). Biofilm control by ionic liquids. Drug Discov. Today.

[117] Miskiewicz A., Ceranowicz P., Szymczak M., Bartuś K., Kowalczyk P. (2018). The use of liquids ionic fluids as pharmaceutically active substances helpful in combating nosocomial infections induced by Klebsiella Pneumoniae new delhi strain, Acinetobacter Baumannii and Enterococcus species. Int. J. Mol. Sci..

[118] Gindri I.M., Palmer K.L., Siddiqui D.A., Aghyarian S., Frizzo C.P., Martins M.A.P., Rodrigues D.C. (2016). Evaluation of mammalian and bacterial cell activity on titanium surface coated with dicationic imidazolium-based ionic liquids. RSC Adv..

[119] Raucci M.G., Fasolino I., Pastore S.G., Soriente A., Capeletti L.B., Dessuy M.B., Giannini C., Schrekker H.S., Ambrosio L. (2018). Antimicrobial Imidazolium Ionic Liquids for the Development of Minimal Invasive Calcium Phosphate-Based Bionanocomposites. ACS Appl. Mater. Interfaces.

[120] Fang C., Kong L., Ge Q., Zhang W., Zhou X., Zhang L., Wang X. (2019). Antibacterial activities of N-alkyl imidazolium-based poly(ionic liquid) nanoparticles. Polym. Chem..

[121] Muñoz-Bonilla A., Fernández-García M. (2018). Poly(ionic liquid)s as antimicrobial materials. Eur. Polym. J..

[122] Garcia I.M., Souza V.S., Souza J.D., Visioli F., Leitune V.C.B., Scholten J.D., Collares F.M. (2020). Zinc-based particle with ionic liquid as a hybrid filler for dental adhesive resin. J. Dent..

[123] Claus J., Jastram A., Piktel E., Bucki R., Janmey P.A., Kragl U. (2021). Polymerized ionic liquids-based hydrogels with intrinsic antibacterial activity: Modern weapons against antibiotic-resistant infections. J. Appl. Polym. Sci..

